# Safety of specific subcutaneous immunotherapy in patients with allergic rhinitis

**DOI:** 10.1186/1939-4551-8-S1-A145

**Published:** 2015-04-08

**Authors:** Mario Henrique Almeida Da Fonseca, Norma Rubini, Albertina Varandas Capelo, Eliane Miranda Da SilvaFernando Samuel Sion, Carlos Alberto Morais De Sa

**Affiliations:** 1 Federal University of the State of Rio De Janeiro, Brazil

## Background

Specific subcutaneous immunotherapy (SCIT) with dust mites is a proven effective procedure in the treatment of allergic rhinitis (AR). However, there is concern about the risk of adverse reactions, especially anaphylactic reactions. The aim of this study was to evaluate the rate of adverse reactions to SCIT with mites in patients with AR in Allergy and Immunology Clinic.

## Methods

We conducted a retrospective, longitudinal study evaluating patients with AR, over the age of three, who were using SCIT with extracts of *D pteronyssinus* (Dp) and/or *B tropicalis* (Bt) (FDA Allergenic). Data were collected through interviews and medical records. The classification of the severity of adverse reactions met the criteria established by the *Allergen Immunotherapy: a Practice Parameter Third Update* (2011).

## Results

100 patients with AR, aged between 3 and 64 (mean = 21.18, SD = 19.89), who received a total of 6,370 applications SCIT, were evaluated. The overall rate of adverse reactions/applications was 0.09% (6), 0.06% (4) being grade I and II. systemic reactions. Three systemic reactions were immediate and one occurred 24 hours after the application. The rate of local reactions/patient was 2% and systemic reactions/patient was 4%. Three patients were using SCIT with Dp/Bt extract and 2 with Dp extract. There was no relationship between the phase of SCIT, association with asthma, severity of rhinitis, serum IgE levels, sex or age with the occurrence of adverse events (p> 0.05).

## Conclusions

In this study, the rate of adverse reactions to SCIT with mites in patients with AR was low, confirming the safety of this procedure. No factor related to an increased risk of adverse reactions was identified, probably due to the small number (6) of observed adverse events observed. The observation of a case of late systemic reaction indicates the importance of counselling patients and caregivers about this possibility.

